# Investigation on urban greenspace in relation to sociodemographic factors and health inequity based on different greenspace metrics in 3 US urban communities

**DOI:** 10.1038/s41370-022-00468-z

**Published:** 2022-08-22

**Authors:** Seulkee Heo, Michelle L. Bell

**Affiliations:** School of the Environment, Yale University, New Haven, CT USA

**Keywords:** Climate change, Environmental justice, Exposure modeling, Health studies.

## Abstract

**Background:**

Study results are inconclusive regarding how access to greenspace differs by sociodemographic status potentially due to lack of consideration of varying dimensions of greenspace.

**Objective:**

We investigated how provision of greenspace by sociodemographic status varies by greenspace metrics reflecting coverage and accessibility of greenspace.

**Methods:**

We used vegetation levels measured by Enhanced Vegetation Index (EVI), percent of greenspace, percent tree cover, percent tree cover along walkable roads, and percent of people living ≤500 m of a park entrance (park accessibility). We considered data for 2008–2013 in Census block groups in 3 US regions: New Haven, Connecticut; Baltimore, Maryland; and Durham, North Carolina. We examined geographical distribution of greenspace metrics and their associations with indicators of income, education, linguistic isolation, race/ethnicity, and age. We used logistic regression to examine associations between these greenspace metrics and age-standardized mortality controlling for sociodemographic indicators.

**Results:**

Which region had the highest greenspace depended on the greenspace metric used. An interquartile range (33.6%) increase in low-income persons was associated with a 6.2% (95% CI: 3.1, 9.3) increase in park accessibility, whereas it was associated with 0.03 (95% CI: −0.035, −0.025) to 7.3% (95% CI: −8.7, −5.9) decreases in other greenspace metrics. A 15.5% increase in the lower-education population was associated with a 2.1% increase (95% CI: −0.3%, 4.6%) in park accessibility but decreases with other greenspace metrics (0.02 to 5.0%). These results were consistent across the 3 study areas. The odds of mortality rate more than the 75th percentile rate were inversely associated with all greenspace metrics except for annual average EVI (OR 1.27, 95% CI: 0.43, 3.79) and park accessibility (OR 1.40, 95% CI: 0.52, 3.75).

**Significance:**

Environmental justice concerns regarding greenspace differ by the form of natural resources, and pathways of health benefits can differ by form of greenspace and socioeconomic status within communities.

**Impact statement:**

Comparisons of exposure to greenspace between different greenspace metrics should be incorporated in decision-making within local contexts.

## Introduction

The world is experiencing rapid urbanization, with 75% of the global population is projected to live in urban regions by 2050 s [1]. Climate change is expected to pose significant health threats to urban populations by contributing to increases in ambient temperature, air pollution, and environmental disasters in urban settings (e.g., flooding, fires at wildfire-urban interfaces). Urban spatial planning for greenery and ecosystem is important in long-term sustainable development for adaptation to climate change [2, 3]. Given the contribution of greenery to mitigation of air pollution and extreme heat [4], management of greenspace is important in balancing distribution, accessibility, and sustainability of natural resources in urban regions to minimize harmful impacts of urbanization and climate change on environment and human health [5, 6].

Greenspace is defined as open land with natural vegetation, although it exists in multiple forms such as parks, urban open spaces, greenery, and street trees [7]. Numerous studies found that greenspace is disproportionately distributed by socioeconomic and other demographic characteristics [8], which is recognized as an environmental justice issue. The reasons for sociodemographic inequality of access to greenspace include history of land development prioritizing communities with higher percent of White and affluent people, histories of racial segregation, and evolution of ideas for using greenspace for leisure and recreation [9]. Marginalized persons or communities such as those who are low income or racial/ethnical minority populations tend to live in more polluted areas and have worse mobility, baseline health status, and access to other health promoting resources [10, 11]. Sociodemographic disparities also exist in burden of disease and mortality [12]. Therefore, ensuring access to public greenspace for these vulnerable persons is important as health benefits from greenspace can be particularly crucial for those with lower socioeconomic status [11]. However, study results are inconclusive regarding the equity of greenspace by sociodemographic subgroups in the US and elsewhere, possibly due to the heterogeneity of how greenspace is defined. For example, a study on 4 US communities (Durham, NC; Austin, TX; Fresno, CA; Minneapolis/St. Paul, MN) found that a combined index of 8 ecosystem service indicators indicated higher exposure to more mixed vegetation and parks in neighborhoods with higher income and education [3]. Another US study found that Black populations were more likely to live closer to urban parks in Baltimore, Maryland [13]. A study in Beijing found that low-income social groups had better access to parks than the general population [14]. Inconsistent results warrant examinations of local conditions or inequity of greenspace [15].

Numerous studies examined the positive effects of exposure to natural greenspace on human health focusing on outcomes such as cardiovascular health, respiratory health, infant growth, obesity, cancer, and kidney diseases [7]. The mechanisms by which greenspace can affect physical and psychological health are complex. Markevych et al. suggested 3 pathways including mitigation (e.g., reducing air pollution and urban heat island effects), restoration (e.g., psychological recovery, stress relief), and instoration (e.g., encouraging physical activities, social connection) [11]. Lachowycz et al. suggested that access to greenspace measured as distance and characteristics of greenspace including amount and quality of greenspace are key factors moderating the use of greenspace and resultant health benefits [16]. Knobel et al. suggested that accessibility is more directly related with urban green spaces and human health pathways than coverage of greenspace [17]. Others hypothesized that short distance to greenspace is important to promote physical activities in greenspace [18]. The amount of greenspace within a region may be crucial for purifying air pollution, controlling urban heat, and providing scenic views, while distance to greenspace may be important for motivating use and visits to greenspace. Greenspace along with walkable streets is important for providing opportunities for interacting with the nature, increasing thermal comfort, and filtering air pollution [19]. Due to various moderating factors in potential mechanisms for how greenspace influences health, it is important to consider various dimensions of greenspace in examining inequity of access to greenspace.

The most commonly applied greenspace metrics in studies of health are greenspace cover and vegetation levels [20]. Both are useful metrics to measure overall density of greenspace, but they do not distinguish among different forms (e.g., trees, parks, forests) and public usability (e.g., privately owned greenspace vs. cemetery). Also, total area of greenspace does not consider accessibility factors such as location of entrance and distance to greenspace. As a result, little is known regarding whether different types of greenspace, and thereby the pathways for health effects of greenspace, differ for socioeconomically disadvantaged or otherwise racially/ethnically marginalized persons. The lack of evidence on accessibility of greenspace compared to that for amount of greenspace calls for examination of associations among health, socioeconomic status and other demographic factors, and distribution of different greenspace metrics.

We analyzed how accessibility and amount (coverage) of greenspace are disproportionately distributed in three US urban regions. Both amounts of and proximity to greenspace are important for equitable provision of greenspace; various GIS measurements for greenspace have led to uncertainties in the results across studies on access to urban greenspace in relation to socioeconomic status [21]. Therefore, we hypothesized that different metrics have different implications for structural environmental racism and sociodemographic inequity. We examined 5 greenspace metrics: vegetation level, amount of greenspace, population living close to a park entrance, tree canopy cover, and tree cover near busy roads. We also examined whether different greenspace metrics along with other environmental exposures (air pollution) have different associations with mortality rates to inform health studies of greenspace in choosing appropriate greenspace indicators for health outcomes. Our study can aid future research and decision-making in urban planning and sustainable development for improving human health and well-being.

## Materials and methods

### Study area

We considered three urban centers: New Haven, CT; Baltimore, MD; and Durham, NC. These cities were selected for this study as we aimed to focus on regions with a range of climate conditions for vegetation growth in different parts of US, with considerations for population size and available data sources for various greenspace indices. The National Centers for Environmental Information has divided the US into nine climatically consistent regions [22]; our study cities are in the Northeast and Southeast regions. The cities have similar overall average greenness, but have differences in the percentages of the population that is low income and people of color and have different population sizes. The cities have variation in greenness, such as a similar overall greenspace level by some metrics (annual median Enhanced Vegetation Index [EVI]: 0.29, 0.29, and 0.33 for New Haven, Baltimore, and Durham, respectively) but differences in greenness by other metrics (e.g., median percent of people living ≤500 m of a park entrance: 20.88 m 16.22, and 8.55, respectively).

The boundary and unit of analysis for these areas were derived from the EnviroAtlas database developed by the US Environmental Protection Agency (EPA) [23], which provided the major greenspace metrics used. EnviroAtlas is an open access tool providing environmental and demographic data in an ecosystem services framework [24]. For EnviroAtlas data, EPA initially derived communities in these 3 areas from the 2010 US Census Bureau’s Urbanized Areas, which include densely settled core Census blocks meeting minimum population density and size (≥50,000 people). Then, EPA identified and included Census block groups (comprising 4–10 Census blocks) with at least ≥50% of their population within the Census Bureau’s Urbanized Area boundary. Even though EnviroAtlas labeled their target communities with the city-level names (e.g., “New Haven“), EnviroAtlas boundaries often contain many municipalities near the target city as these EnviroAtlas boundaries are based on Census Urbanized Areas rather than the city boundaries. Supplemental Table S1 provides the municipalities included in the three urban areas, hereafter referred to as “Durham”, “New Haven”, and “Baltimore” to represent the regions [23]. The number of Census block groups included in the EnviroAtlas boundaries were 444, 1648, and 193 for New Haven, Baltimore, and Durham, respectively. The number of ZIP Code Tabulation Areas (ZCTAs), which are the generalized representations of the US postal service Zip code defined by the US Census Bureau and the unit of observation for several statistical analyses of our study described in the next sections, were 32, 124, and 13 for New Haven, Baltimore, and Durham, respectively. The 3 study areas are shown in Fig. S1. Due to the difference in the smallest available spatial units of our socioeconomic variables (e.g., block group level) and mortality rates (e.g., ZCTA), our statistical analysis had varying spatial units of observation as described in the analysis section.

### Greenspace metrics

#### Satellite remote sensing data for vegetation index

Vegetation levels were measured by using EVI from the Moderate Resolution Imaging Spectroradiometer (MODIS) product MOD13Q1 by the NASA’s Earth Observing System for 2008–2013 to match the metrics of EnviroAtlas. The MOD13Q1 product is a 16-day composite image at 250 m resolution providing EVI calculation based on near-infrared radiation minus visible radiation divided by near-infrared radiation plus visible radiation. The index ranges between −1 to 1 with higher values indicating more dense vegetation. The population-weighted average EVI for each ZCTA was calculated;

$$EVI_{i,t} = \mathop {\sum}\limits_1^i {\left( {EVI_{ci,t} \times POP_c} \right)/POP_i}$$

where *EVI*_*i,t*_ is the EVI value of ZCTA *i* on date *t*, *EVI*_*ci,t*_ is the EVI value of block group c of ZCTA _*i*_ on date *t*, *POP*_*c*_ is the population of block group *c*, and *POP*_*i*_ is the population of ZCTA *i*. Populations for each block group and ZCTA for were obtained from the American Community Survey (ACS) 5-year data providing data for 2008–2012 [25]. EVI of each block group was estimated by averaging the pixel values within and surrounding the block group boundary. By averaging all 16-day population-weighted average EVI in 2008–2012, we calculated an average population-weighted EVI value for each ZCTA.

As vegetation follows a seasonal cycle with the highest amount of green during the growing season, we also calculated seasonal EVIs for May–October, which is the consistent growing season of our study areas based on the Plant Hardiness Zone Map of the US [26], for each block group and ZCTA.

#### Percent greenspace

We obtained data for percent greenspace for each census block group from the EnviroAtlas database [24]. Percent green space refers to the percentage of land covered by vegetation or greenspace (trees, lawns, gardens, crop land, forests, wetlands). This index was derived from land cover data at 1-m resolution for 2008–2013 through remote sensing methods by the National Land Cover Dataset (NLCD) [27].

#### Percent tree canopy cover

Percent tree cover was obtained from the EnviroAtlas database. EnviroAtlas estimated percent tree cover for each block group using NLCD data for 2008–2013. This metric illustrates the percent of total land covered by trees including street trees, parks, forests, and single trees.

#### Tree cover along walkable roads

Percent of tree cover along walkable roads for each block group was obtained from the EnviroAtlas database, which used NLCD data for 2008–2013. EPA calculated this metric using tree cover in an 8.5-meter strip from the estimated road edge from the GIS data provider company NAVTEQ’s road centerlines dataset. To identify potentially walkable streets, EPA included only roads with a speed limit <55 miles/h in the estimation using the dataset from NAVTEQ, a commercially available road dataset [28]. Calculation for these metrics was performed using 2010 US census block group boundaries within EnviroAtlas boundaries.

#### Accessibility to park entrance

The EnviroAtlas database provided an index of population living ≤500 m from a park entrance (state, county, or local park) for each census block group [29]. Only parks within a 5 km buffer around EnvironAtlas boundaries (Fig. S1) were included. Using multiple sources of satellite imagery data and the best available road network dataset, EPA designated the park entrance for every 0.50 kilometer along the border of a given park that was open to the street to estimate this index. Distance was based on walking distance to the nearest park entrance from every point along the roads in each census block group. Using this index and population estimates in each census block group from ACS 5-year data [25], we calculated population-weighted percentage of population living ≤500 m from a park entrance for each ZCTA. As we refer to this index as accessibility to park entrance, this index does not represent park size.

### Sociodemographic characteristics

We obtained demographic indicators at the census block group from EPA’s EJSCREEN database (EPA) [30]. We used percent of population in households with household income ≤ twice the federal poverty level, percent of people of color (e.g., percent of individuals with a race other than White alone and/or with ethnicity as Hispanic/Latino), percent ≥25 years with less than high school education, percent of living in linguistically isolated households (e.g., household in which all members age 14 years and older speak a non-English language and also speak English less than “very well”), percent of people under the age of 5 years, and percent of people over the age of 64 years. Also, we calculated population density of each ZCTA using ACS 5-year estimates for population for 2008–2012.

### Air pollution

We obtained a 1 km resolution estimation modelled data for annual fine particulate matter (PM_2.5_) and ozone (O_3_), which are the two criteria air pollutants that have shown relatively less declines of annual average concentrations since the 1970s compared to the other criteria air pollutants in the US according to previous literature [31], for the year 2012 from the Socioeconomic Data and Application Center [32]. Both of these pollutants have substantial public health burden [33]. These values were estimated with generalized additive model assembling daily predictions from machine learning models [34]. The machine learning models incorporate satellite data, meteorological variables, land use variables, elevation, and chemical transport modeling. Annual estimates were based on averaging daily predictions for each year and grid cell. We calculated average annual concentration of PM_2.5_ and O_3_ for each block group in 2012 by averaging pixel values within and surrounding each census block group boundary. Then, we calculated population-weighted concentration of PM_2.5_ and O_3_ for each ZCTA.

### Mortality data

We examined whether regions with lower mortality risk have higher exposure to greenspace using different greenspace indicators and mortality data from 2005–2017. Mortality data were obtained from the NC, MD, and CT state health agencies. Spatial scales of the raw mortality data differed across the study states in this research. The individual-level records of mortality in CT provided census block group of residence for decedents and the individual-level records of mortality in NC provided coordinates of residence for decedents. Meanwhile, aggregated death counts at the ZCTA level were obtained for MD. We calculated annual age-standardized mortality rate (SMR) for all-cause death (International Classification of Diseases [ICD] version 10 code: A00–R99), circulatory disease (I00–I99), respiratory diseases (J00–J99), cancers (C00–D49), renal disease (N00–N39), and mental disorders (F01–F99) in each year and ZCTA. Then, annual SMRs for each cause of death were averaged through 2005–2017 for each ZCTA. While the spatial scales differed across the raw mortality datasets among the study areas, the spatial unit of regression analyses for SMR in relation to greenspace and socioeconomic/demographic variables was consistent (e.g., ZCTA) among the states, as described in the next section.

### Statistical analysis

We compared exposure to greenspace using 5 greenspace metrics among quartile groups of sociodemographic indicators and box plots. We categorized the block groups into 4 quartiles separately for sociodemographic variables, individuals <5 years, and individuals >64 years. This categorization for block groups was based on quartile values of each indicator within each study region (i.e., New Haven, Baltimore, Durham). One-way analysis of variance (ANOVA) analysis was applied to examine differences in greenspace across quartiles of each indicator.

We tested the non-linearity of the exposure-response relationships for the greenspace metrics, sociodemographic indicators, and the SMRs using generalized additive models. The relationship curves indicated linear relationships for almost all variables justifying a linear regression analysis for the associations among the variables. As a result, we used generalized linear regression modeling to estimate associations for greenspace with air pollution and sociodemographic indicators, separately to each greenspace metric:

$$G_{m,i} = \alpha + \beta _1X_{1i} + \ldots + \beta _kX_{k,i} + \varepsilon$$

where *G*_*l,i*_ = *m*-th greenspace metric for block group *i*, *β*_*k*_ = regression coefficient linking explanatory variable *x*_*j*_ (*j* = 1, …k), and *ε* = error. Explanatory variables included percent people of color, percent with low income, percent living in linguistically isolated households, percent of people ≥25 years with education less than high school, percent of individuals <5 years, percent >64 years, and population density in this model. Main results were presented by the changes in the level of greenspace metrics for interquartile range (IQR) increase in explanatory variable. We used IQR as a standard unit increase to present results as the scales and ranges differed across the explanatory variables (i.e., percentages of socioeconomic and demographic variables vs. population density) to report the associations. We adjusted for coordinates of the centroid of each block group and an indicator variable for each study region. We also applied regression models separately to each study region. As the estimates were available at the block group level for the greenness metrics and sociodemographic variables, the spatial unit of these regression models was census block group.

We conducted logistic regression analyses including SMRs a binary variable of cause-specific mortality rate over the 75th percentile of SMR in the ZCTAs in each study region (i.e., ≥75th percentile or <75th percentile of SMR). As the smallest available spatial unit of the mortality rate variables in this study was ZCTA, the spatial unit for these logistic regression models was ZCTA. Models were separately applied to each greenspace metric aggregated at the ZCTA level. Covariates included annual mean PM_2.5_, annual mean O_3_, percent low-income, percent of people of color, percent linguistically isolated households, percent ≥14 years with less than high school education, percent <5 years, and percent >64, for each ZCTA. Covariables were calculated with population-weighting. All models adjusted for coordinates of the centroid, an indicator variable for region, and population density of each ZCTA.

We also conducted analyses using the EVI levels during the growing season (May–October) for the associations between EVI and sociodemographic indicators. This approach using the seasonal EVI levels was also applied to the sensitivity analysis of logistic regression models for the associations between EVI and the binary variable of SMRs (≥75th percentile).

In a sensitivity analysis, we considered matching the time period of average SMRs in the study ZCTAs to the period of greenspace metrics (i.e., 2008–2013).

We checked the normality of residuals of all regression models in this study and the normality of residuals was satisfied.

## Results

Table 1 shows descriptive statistics (mean, standard deviation, Q1, Q3). Durham showed relatively higher percentage of low income, people of color, linguistic isolation, and low education compared to the other study areas. Which study region had the highest greenspace depended on the greenspace metric used. Average percent greenspace was highest in Durham and lowest in Baltimore. Averages of annual EVI (0.32) and percent tree cover (56.85%) were highest in Durham. Average of percent of people living ≤500 m of a park entrance and percent tree cover along walkable roads were highest in New Haven. Annual all-cause SMR was highest in Baltimore (mean 81.9 deaths per 100,000 people).

**Table 1:** Descriptive statistics of sociodemographic variables, greenspace metrics, and mortality rate.

Variable	New Haven, CT				Baltimore, MD				Durham, NC			
	Mean	SD	Median	Q1-Q3	Mean	SD	Median	Q1-Q3	Mean	SD	Median	Q1-Q3
Sociodemographic indicator^a^												
Percent low income (%)	26.53	21.57	19.37	9.74–39.23	28.71	22.53	22.62	10.45–43.07	41.18	25.75	39.70	19.82–59.10
Percent people of color	33.32	30.52	20.93	9.55–55.04	47.99	34.85	40.01	16.21–86.03	51.13	28.02	49.22	24.85–76.35
Percent linguistically isolated	4.84	6.82	2.58	0.00–6.73	2.07	4.13	0.00	0.00–2.66	6.00	8.17	3.26	0.00–8.04
Percent of people with less than high school education	11.89	10.65	8.69	4.25–15.92	13.94	12.16	10.93	4.44–20.39	13.26	12.89	8.85	2.56–20.59
Percent of population <5 years	5.30	3.66	4.77	2.82–7.19	6.01	4.30	5.55	2.94–8.33	6.61	4.42	6.38	3.49–9.39
Percent of population >64 years	15.24	9.21	13.95	8.74–19.44	13.53	9.72	11.78	7.16–17.71	10.34	8.29	8.34	4.93–13.58
Population density (person/km^2^)	2033.5	2342.4	1189.8	407.1–2768.4	3127.3	3168.6	2084.1	911.4–4307.0	1203.0	1023.3	915.0	550.8–1543.7
Greenspace metrics^a^												
Annual EVI	0.28	0.07	0.29	0.23–0.34	0.27	0.08	0.29	0.23–0.33	0.32	0.04	0.33	0.30–0.35
EVI in growing season (May–Oct.)	0.40	0.10	0.41	0.33–0.48	0.36	0.11	0.38	0.29–0.36	0.40	0.06	0.41	0.37–0.45
Percent greenspace	67.70	1.88	70.89	54.88–83.09	59.08	24.72	62.78	42.94–78.94	71.40	13.14	72.44	65.01–81.43
Percent tree cover	48.19	19.17	48.23	32.59–61.73	36.46	20.00	35.45	19.93–50.59	56.85	14.06	59.83	48.09–67.24
Percent of people living ≤500 m of a park entrance	33.64	33.52	20.88	4.09–58.66	32.03	35.63	16.22	0.00–60.57	20.89	27.56	8.55	0.00–33.28
Percent tree cover along walkable roads	41.31	16.67	39.43	29.12–52.67	30.55	17.47	27.77	17.04–41.82	36.47	14.72	35.66	25.13–43.85
Mortality rate (person/10,000 population)^b^												
All-cause	65.04	24.97	63.67	54.51–73.57	81.88	87.08	70.12	59.51–89.75	53.53	11.96	53.80	45.20–62.90
Circulatory disease	20.00	7.26	19.57	17.15–22.75	29.81	42.97	23.59	19.25–32.34	15.92	3.80	15.70	12.70–19.10
Respiratory disease	6.41	2.34	6.35	5.23–7.48	7.23	4.89	6.70	5.25–8.69	5.18	1.43	5.30	3.80–5.80
Cancer	17.38	6.30	17.55	13.65–19.38	21.48	21.96	19.02	15.41–23.13	14.30	2.66	14.70	11.90–15.10
Renal disease	1.97	1.19	1.73	1.19–2.56	1.77	0.99	1.70	1.17–2.29	1.85	0.74	1.70	1.40–2.10
Mental disorders	0.33	0.53	0.27	0.12–0.36	6.33	13.06	4.85	3.84–6.10	0.40	0.16	0.40	0.30–0.50

Note. ^a^Census block group level. ^b^ZCTA level.

**Fig. 1:**
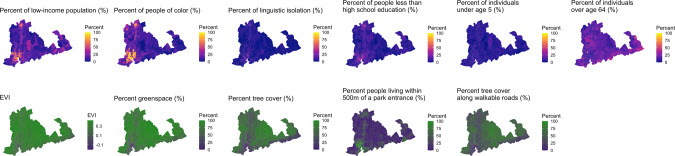
Map of sociodemographic indicators and greenspace metrics in New Haven, CT. Note. EVI: Average EVI values of all days in the years 2008–2013.

**Fig. 2:**
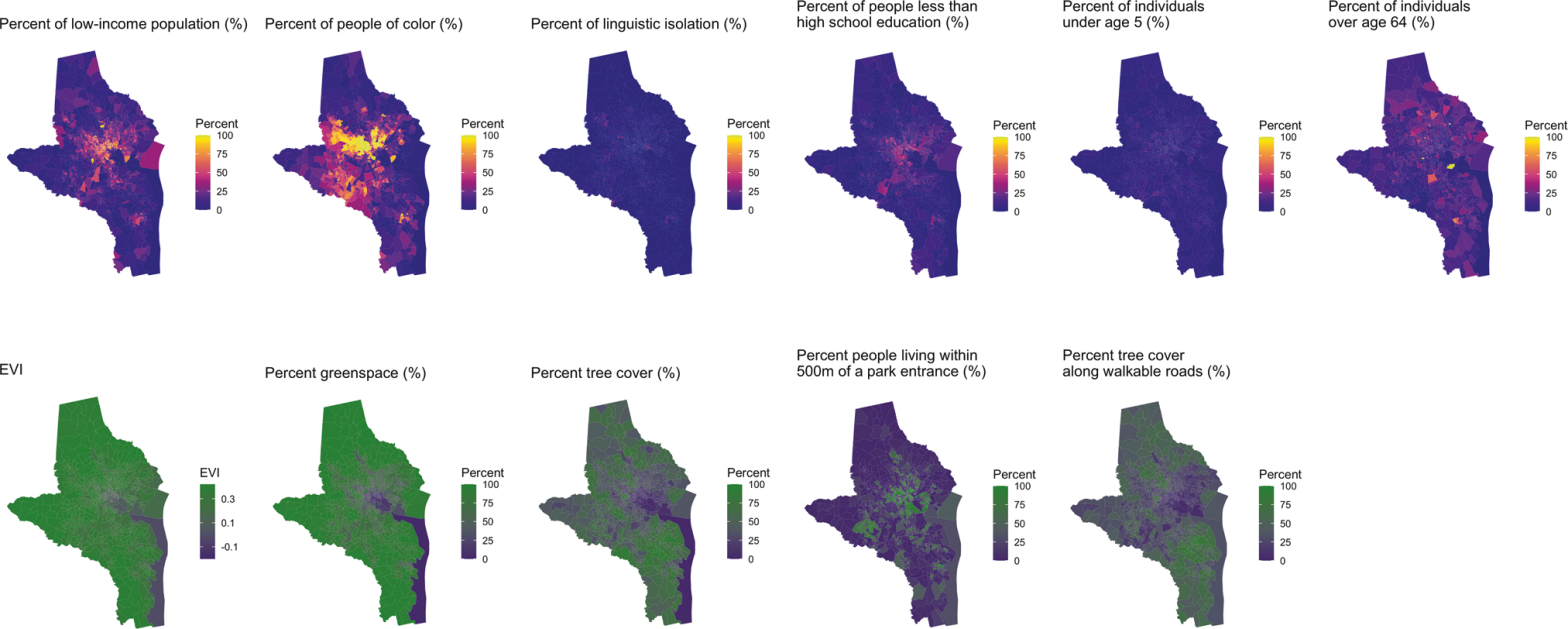
Map of sociodemographic indicators and greenspace metrics in Baltimore, MD. Note. EVI: Average EVI values of all days in the years 2008–2013.

**Fig. 3:**
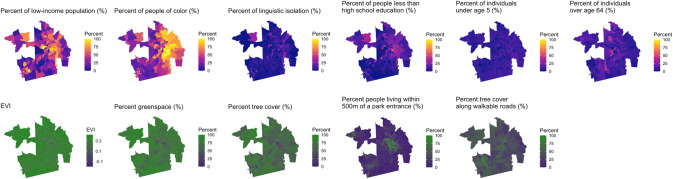
Map of sociodemographic indicators and greenspace metrics in Durham, NC. Note. EVI: Average EVI values of all days in the years 2008–2013.

Figures 1–3 illustrates patterns of sociodemographics and greenspace by block group for the study regions. Populations with low education, low income, and higher percentage people of color were higher in the central areas of Baltimore and Durham. They were also higher in the most populated areas near southern New Haven. In New Haven, the greenspace metrics, except for percent of people living ≤500 m of a park entrance, corresponded with higher-income, higher percent of White persons, less linguistically isolated, and more educated communities. In all three study regions, percent of people living ≤500 m of a park entrance showed contrasting spatial distributions compared to the other greenspace metrics. Vegetation level and greenspace coverage based on all 5 metrics were higher in peripheral suburban areas than the most populated communities in each study region, whereas park entrances were more accessible to residents in the most populated areas compared to the peripheral regions.

**Fig. 4:**
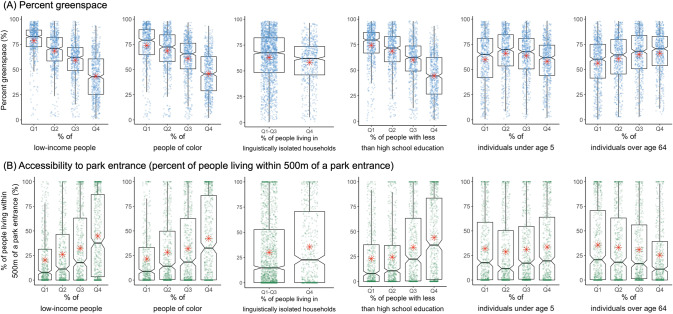
Comparison of exposure to greenspace among quantile groups of percent of people with low income, people of color, lower education, linguistic isolation, and individuals less than age 5 or over age 64 years (*n* = 2285). (**A**) Percent greenspace; (**B**) accessibility to park entrance in the study block groups. Note. Red asterisk indicates the average within each group. Green and blue points indicate value of greenspace metrics in each block group.

We examined how level of greenspace varied by community characteristics. Figure 4 shows the distribution of greenspace among quantiles of census block groups by percent of people with low income, people of color, lower education, and individuals <5 or >64 years. The percentage of linguistically isolated households was less than 10.0% in about 92.1% (*n* = 2105) of the census block groups considered in this study. Due to this skewed distribution, percentages of people living in linguistically isolated households were categorized into two groups (i.e., Q1-Q3, Q4). Percent greenspace was higher in block groups with lower percentages of people of low income, people of color, and people with lower education (Fig. 4). ANOVA analysis showed significant differences in percent greenspace across quartiles of sociodemographic variables (*p*-value < 0.001). In contrast, accessibility to park entrances was higher in block groups with higher percent of disadvantaged populations (*p*-value < 0.001) except for percent of individual <5 years (*p*-value = 0.116). Exposure to greenspace and park accessibility did not show linear correlations with percent of individuals <5 years based on the boxplot (Fig. 4). Individuals >64 years showed higher exposure to greenspace but less access to park entrances. Annual EVI, percent tree cover, and percent tree cover along walkable roads showed similar exposure patterns in relation to sociodemographic factors with the pattern for percent greenspace (Fig. S2).

Using regression analysis, we examined associations between greenspace metrics and sociodemographic indicators (Table 2). An IQR increase in low-income persons (IQR=33.6%) was negatively associated with annual EVI, percent greenspace, percent tree cover, and percent tree cover along walkable roads, and was positively associated with percent of people living within 500 m of a park entrance. For example, a 33.6% increase in low-income persons was associated with 6.2% increase (95% CI: 3.1, 9.3) in percent of people living ≤500 m of a park entrance. This positive association for park access was consistent in regression analyses separately applied to each study area (Table S4). A 33.6% increase in low-income persons was associated with 9.3% (95% CI: 1.9, 16.6), 5.1% (95% CI: 1.2, 9.1), and 9.1% (95% CI: 2.1, 16.0) increase in access to park entrance in New Haven, Baltimore, and Durham, respectively. An IQR increase in the percent of persons who were people of color was significantly and negatively associated with percent greenspace (2.0% decrease, 95% CI: −3.7–0.4) and percent tree cover along walkable roads (2.6% decrease, 95% CI: −4.1, −1.0) (Table 2). An IQR increase (15.5%) in percent of people with less than high school education showed positive associations for percent of people living ≤500 m of a park entrance, in contrast to results for the other 4 greenspace metrics. Results for all study areas indicated that neighborhoods with higher percentage of population age <5 or >64 years had reduced access to park entrances compared to other neighborhoods, whereas they had higher land cover by greenspace or tree coverage. However, such negative associations were only found in Baltimore when study areas were analyzed separately. Overall, the different associations for access to park entrance compared to the other greenspace metrics were found from both regression models for all areas and separate models for each area.

**Table 2:** Regression analysis for greenspace metrics and sociodemographic indicators in Census block groups in 3 study regions: Changes in greenspace metrics for an IQR increase in explanatory variables (*n* = 2,285).

Variable	Beta (95% CI)				
	EVI (annual)	Percent greenspace	Percent tree cover	Percent of people living within 500 m of a park entrance	Percent tree cover along walkable roads
Percent of persons who are people of color (IQR = 64.7)	0.004 (−0.003, 0.010)	−2.0 (−3.7, −0.4)*	−0.7 (−2.3, 1.0)	0.4 (−3.3, 4.0)	−2.6 (−4.1, −1.0)*
Percent of low income (%) (IQR = 33.6)	−0.030 (−0.035, −0.025)*	−7.3 (−8.7, −5.9)*	−5.8 (−7.3, −4.4)*	6.2 (3.1, 9.3)*	−3.1 (−4.4, −1.7)*
Percent of linguistic isolation (IQR = 3.8)	0.000 (–0.002, 0.002)	0.2 (−0.2, 0.7)	−0.1 (−0.5, 0.4)	−0.3 (−1.3, 0.7)	−0.1 (−0.5, 0.4)
Percent of people with less than high school education (IQR = 15.5)	−0.015 (−0.019, −0.011)*	−3.6 (−4.7, −2.5)*	−4.0 (−5.1, −2.9)*	2.1 (−0.3, 4.6)	−5.0 (−6.1, −4.0)*
Percent of population under 5 years (IQR = 5.3)	0.006 (0.004, 0.009)*	1.5 (0.7, 2.3)*	0.9 (0.2, 1.7)*	−2.3 (−4.1, −0.6)*	0.5 (−0.3, 1.2)
Percent of population over 64 years (IQR = 10.7)	0.002 (0.000, 0.005)*	0.4 (−0.3, 1.1)	0.8 (0.1, 1.5)*	−2.6 (−4.2, −1.0)*	1.7 (1.0, 2.3)*
Population density (people/km^2^) (IQR = 3070.6)	−0.035 (−0.037, −0.032)*	−13.4 (−14.1, −12.6)*	−8.2 (−8.9, −7.4)*	11.2 (9.6, 12.9)*	−4.6 (−5.3, −3.9)*

Note. All models were adjusted for the centroids of each block group and an indicator variable of study regions.

*Significant at a significance level of 0.05.

In sensitivity analysis using the EVI levels in the growing season, the results for the associations with sociodemographic indicators (Table S6) were consistent with the results from the model using the annual EVIs shown in Table 2.

We examined associations between mortality (SMRs) and greenspace, adjusted for sociodemographic indicators and air pollution (Table 3). The OR of having an all-cause SMR ≥ the 75th percentile mortality rate in relation to different greenspace metrics. SMRs were significantly lower in ZCTAs with higher greenspace metrics except for average annual EVI and percent of people living within 500 m of a park entrance. SMRs were higher in ZCTAs with higher park accessibility (OR = 1.40, 95% CI: 0.52, 3.75), although the results were not significant. There was a difference between the annual EVI levels and EVI levels in the growing season in relation to SMRs. Annual EVI levels were positively associated with the odds of SMRs ≥75th percentile and seasonal EVI levels were negatively associated with these odds. However, neither annual and seasonal EVIs were significantly associated with the all-cause SMRs.

**Table 3:** Odds ratios of having an all-cause standardized mortality rate (SMR) ≥ the 75th percentile mortality rate in relation to greenspace metrics for the study ZCTAs (*n* = 169).

Variable	OR (95% CI)
Annual EVI (IQR = 0.08)	1.27 (0.43, 3.79)
EVI in growing season (IQR = 0.12)	0.82 (0.26, 2.62)
Percent greenspace (%) (IQR = 28.1)	0.02 (0.00, 0.13)*
Percent tree cover (%) (IQR = 26.6)	0.01 (0.00, 0.07)*
Percent of people living within 500 m of a park entrance (%) (IQR = 21.1)	1.40 (0.52, 3.75)
Percent tree cover along walkable roads (%) (IQR = 19.6)	0.08 (0.02, 0.41)*

Note. All models were adjusted for the centroids of each block group, an indicator variable of study regions, annual mean PM_2.5_ and O_3_ concentrations, percent of low-income people, percent of people who are people of color, percent of linguistic isolation, percent of people with less than high school education, percent of population under 5 years, percent of population over 64 years, and population density.

*Significant at a significance level of 0.05.

Results for cause-specific SMRs associated with greenspace metrics are shown in Fig. S3 and Table S3. The odds of having SMR ≥ 75th percentile were significantly lower in ZCTAs with higher percent greenspace and percent tree cover within the ZCTA for annual SMR from circulatory diseases, respiratory diseases, cancers, renal diseases, and mental disorders. EVI (both annual and seasonal averages) and percent of people living ≤500 m of a park entrance were not significantly associated with any cause-specific SMRs. The sensitivity analysis using the all-cause SMRs in the study ZCTAs in the period matching with the greenspace metrics (i.e., 2008–2013) showed robust results for the impacts of each greenspace metric on the SMRs (Table S9).

## Discussion

To date, while research has indicated health benefits of greenspace, less is known about the heterogeneity of associations between greenspace metrics and health in urban regions, especially with respect to different types of greenspace. As there are substantial differences in the definition of coverage of greenspace, vegetation density, and proximity to greenspace, health studies generally analyzed associations between a single greenspace-related index and mortality. The lack of comparison of mortality risks across multiple greenspace metrics may contribute to inconclusive findings for socioeconomic inequity of greenspace and health effects of greenspace. We examined distribution of greenspace, considering different metrics of greenspace, to identify disparities and thereby add evidence on neighborhoods where health benefits of greenspace can be further improved. Our examination of relationships between greenspace metrics and sociodemographic indicators had high agreement in all 3 US urban areas. Our results showed that land covered by greenspace was more prevalent in suburban regions with higher percentage of affluent people, while public parks were more accessible from core city areas where income level was lower and percent of persons who are people of color was higher. This is consistent with our hypothesis that different greenspace metrics have different implications for inequity of greenspace. Our results are similar to previous systematic review studies suggesting that racial/ethnic minority groups have slightly better access to parks in terms of proximity across several global northern countries and China [12, 35, 36].

Visits to greenspace and physical activities in greenspace increase with decreases in distance and with more formal forms of greenspace such as urban parks [37]. Urban parks can provide versatile facilities and services to meet public needs, such as exercise, relaxation, aesthetic values, and social cohesion, compared to other forms of greenspace such as unstructured vegetated areas [38]. Different greenspace metrics, commonly used in public health research, may reflect different pathways for health benefits. Some metrics may reflect ecology, while others may indicate more direct factors (e.g., attractiveness of greenspace, accessibility) for healthier lifestyles and behaviors. For example, percent greenspace and tree cover may affect human health through visiting and viewing of greenspace that include possible pathways for physical activity and different influences on stress reduction compared to other forms of greenspace. Reduction in air pollution and urban heat by forest and vegetation is also a potential pathway to improve health [19]. Tree canopy cover along walkable roads can directly affect health by reducing traffic-related air pollution levels [19]. Also, metrics such as greenspace cover or EVI reflect overall greenness, but obscure variation in types of greenspace such as forests versus urban parks and do not reflect accessibility or spatial segmentation of greenspace. Thus, these metrics are less informative for understanding how the vegetated areas relate to potential mechanisms for health. Park accessibility based on proximity to entrances may reflect more ‘usable’ nature infrastructures. Consideration for varying greenspace measurements can contribute to understanding the magnitude of contributions of various mechanisms to health, which is needed for planning effective interventions [39]. In studies examining socioeconomic and health inequity in relation to greenspace, efforts are needed to provide more comprehensive understanding of varying dimensions of greenspace inequity including accessibility and coverage, which are determinants of visits and use of greenspace.

Interventions for promoting use of parks may diminish health inequities in core areas of urban cities where coverage of greenspace is limited compared to peripheral suburban regions. We found that associations between park accessibility and mortality rates were notably different from associations between mortality and the other greenspace metrics reflecting coverage of greenspace. Mortality rates were higher in ZCTAs with higher park accessibility. Similarly, a recent study found that density of greenspace defined as availability showed significant inverse associations with mortality, whereas proximity to greenspace defined as accessibility was not associated with mortality in the United Kingdom [40]. The lack of beneficial contribution of park accessibility to SMRs in our results warrants further research and may suggest that the parks in our study regions might not fully provide sufficient opportunities for visiting or services motivating physical activities. Safety and attractiveness are significant factors affecting residents’ visits to greenspace or parks, along with other features such as convenience (e.g., parking, public transportation), cost, and crowding. Another possibility is that the size of parks in the highly populated areas is not sufficient to attract visitors and stays compared to larger parks. Connectivity to parks through transportation, which has not been examined in our study, could also affect the use of parks.

Vulnerable communities would benefit from further access to greenspace in park provisions. Parks provide spaces for socialization and physical activities for socioeconomically disadvantaged people who cannot afford other options for exercise such as fitness centers [12]. Studies have suggested that greenspace has therapeutic effects on mental health disorders and cardiovascular diseases [20, 41]. In our study, park accessibility was lower in block groups with higher percentage of persons >64 years, whereas it was higher in block groups with low income, less education, linguistic isolation. Research may be needed for investigating whether health benefits of greenspace exist for mental and neurological disorders and for cognitive health in communities with a higher population of the elderly.

Studies have found lower availability of greenspace for communities with lower socioeconomic status and more people of color across the US [42], but these patterns are inconsistent. For example, previous studies in Maryland showed that Black and low-income persons have a higher number of parks [43], while large size park were more likely to be closer to White communities [13, 42]. Investigation on reasons for this discrepancy is yet limited. One reason for higher access to parks among people of color in our study regions could be the history of demographic, geographic patterns as Whites migrated from urban areas to more racially homogeneous suburban regions, whereas African Americans resided in more urban areas after the White flight in 1960–1970s in US cities [44]. Red-lining and structural racism have substantially impacted sociodemographic patterns of communities in urban areas [45], which in turns affects exposure to greenspace. It is important to understand political drivers related to historical planning in each location as they influence the capability of local movement and interventions for equitable distribution of greenspace. We note that an integration of disciplines including from social sciences, urban planning, exposure science, and public health may be critical for research to best inform decision-making. We urge more attention to both spatial and social contexts of access to greenspace as we attempt to increase equitable provision of greenspace for disadvantaged populations in urban settings. Furthermore, the integration of all affected populations in decision-making (i.e., procedural justice) should be ensured in urban greening [46, 47].

We did not address the demands for equal greenspace by the residents. We also did not have information on visits to greenspace and how visits differed by subpopulation with respect to frequency, activity at greenspaces, or type of greenspace visits. Even though park accessibility was higher in neighborhoods with more socioeconomically disadvantaged persons, there may exist current park congestion and high demand for more attractive and safer park areas [48]. Amenities, safety, and aesthetics of greenspace are also important aspects in decision-making as these features affect likelihood of visiting the greenspace [17, 49]. Furthermore, designs and structure of greenspace that are more attractive for promoting physical activity may differ by cultures and regions [37]. Findings for proximity to parks should be integrated with investigation on quality of local parks and demand for more parks by the residents in future studies, incorporating direct input from residents to best meet community needs.

Changes in environments and land cover in urban regions are occurring rapidly [50]. Although our study provides empirical evidence of discrepancy of geographical patterns of various greenspace metrics, the data for greenspace and sociodemographic indicators need updates for the growth or deforestation of greenspace in communities and more details on types and features of greenspace to be considered in decision-making process. Researchers should identify stakeholders’ views regarding greenspace for the promotion of human health in their communities and create data collection systems for relevant indicators.

Our study has several strengths. Our analysis provides a unique approach to assess multiple types of greenspace exposure that can be linked to the broader framework of urban health and considers three urban areas with different characteristics of greenspace. A limited number of studies have examined inequity of greenspace using various greenspace indicators, and studies examining greenspace provision with a single metric often reached contradictory results on which types of subpopulations or communities were exposed to higher levels of greenspace. This is important as different measurements of greenspace may reflect different health effects and pathways that influence health [51]. We also used multiple sociodemographic indicators at a fine spatial resolution that are important in environmental justice to examine inequity of greenspace.

This study also has limitations. First, this is a cross-sectional study so causality between explanatory variables and greenspace metrics is not established. Rather than aiming to verify the associations between health and greenspace, we aimed to identify whether sociodemographic inequity exists for different exposure measurements of greenspace including by different type of greenspace. Second, we did not have individual-level information of location of residence relating to each death so the exposure measurement for greenspace was not available for finer scales (e.g., buffers for an event point) than Census block group. The size of ZCTAs are approximate to neighborhoods but may be too large to fully reflect microenvironment of urban greenspace, especially in peripheral suburban regions as shown in our spatial analysis. Also, as we used aggregated data for spatial units, the modifiable areal unit problem (MAUP), which refers to a phenomenon that alternative ways of aggregation of spatial units lead to variations in observations, may affect our results. Third, we could not incorporate the full spectrum of characteristics of greenspace including further variation of greenspace type, use of greenspace, nature of activity, etc. Further, although we examined proximity to greenspace in relation to sociodemographic factors, we could not analyze how various subpopulations may visit, use, or perceive greenspace differently. Fourth, greenspace exposure was examined focusing on two race/ethnicity categories, White and people of color, as we used the percent people of color obtained from the EJSCREEN of the EPA. This EJSCREEN index aggregated the percentage of persons whose racial status was other than White-alone and/or ethnicity was Hispanic or Latino into a single group [30]. There may be differences in racial/ethnical distribution among people of color across our three study regions and therefore the racial/ethnic group that least benefited from exposure to greenspace and their spatial distribution may differ. Considering the percentage of Black people instead of all people of color in the model analyzing the association between sociodemographic variables and greenspace showed consistent results with the main results (Table S7). However, further investigation is warranted for racial/ethnical differences in greenspace exposure and health benefits as various populations have different characteristics and experiences with structural racism. Finally, we did not consider green gentrification and how it affected the communities over time as our study design was cross-sectional. Green gentrification may hinder access to high-quality parks and organized open spaces in urban regions for socioeconomically disadvantaged people due to increased land price [39] and may lead to changes in the sociodemographic composition of communities [52].

In summary, we examined inequity of greenspace in 3 US urban areas with different metrics of greenspace, reflecting different aspects of the urban green environment. Exposure to greenspace and vegetated areas was higher in suburban regions where more affluent populations reside, whereas proximity to park entrances was higher in core urban regions where percent of people with low income, people of color, and people with lower education was higher. This discrepancy between the park accessibility and the coverage of greenspace indicates that actual interaction with nature and associated health benefits may vary across communities by geographical locations and sociodemographic factors, and highlights the need to consider multiple forms of greenspace. Decision-makers should consider the provision of different forms of greenspace and their potential health benefits to the communities within local contexts, incorporating the needs and goals of various subpopulations within communities.

## Supplementary information

Supplementary materials

Reporting Checklist

## Data Availability

The datasets generated or analyzed for greenspace during the current study are available in the EnviroAtlas Data repository available at [https://www.epa.gov/enviroatlas/forms/enviroatlas-data-download]. The datasets analyzed for sociodemographic variables are available in the EJSCREEN repository available at [https://www.epa.gov/ejscreen/download-ejscreen-data]. The mortality datasets generated during and/or analyzed during the current study are not publicly available as the data include identifiable and credential information, and the data can be requested for research purposes from relevant public health sectors in the study regions.
