# Supplementary material for: Investigation on urban greenspace in relation to sociodemographic factors and health inequity based on different greenspace metrics in 3 US urban communities
Source: J Expo Sci Environ Epidemiol. 2022 Aug 22;33(2):218–28. doi: 10.1038/s41370-022-00468-z (PMC10005950; doi:10.1038/s41370-022-00468-z)
Supplement: Supplementary file 1 — Supplementary materials [file 41370_2022_468_MOESM1_ESM.docx]

<Supplementary materials>

Investigation on urban greenspace in relation to sociodemographic factors and health inequity based on different greenspace metrics in 3 US urban communities

Seulkee Heo^1^, Michelle L. Bell^1^

^1^School of the Environment, Yale University, New Haven, CT, USA

**Contents**

Table S1. Municipalities included in the 3 urban areas, as defined by EPA’s EnviroAtlas.

Table S2. Definitions and data sources for the exposure and outcome variables and covariates.

Table S3. Odds ratios of SMR for being ≥the 75^th^ percentile mortality rate, in relation to greenspace metrics (*n*=169).

Table S4. Regression analysis for greenspace metrics and sociodemographic indicators in Census block groups in 3 study regions, separately.

Table S5. Odds ratios of all-cause standardized mortality rate (SMR) for being ≥the 75th percentile mortality rate in relation to sociodemographic variables in ZCTAs (n=169) from the model considering each greenspace metric.

Table S6. Regression analysis for EVI in the growing season and sociodemographic indicators in Census block groups in 3 study regions (n=2,285).

Table S7. Sensitivity analysis of greenspace metrics and sociodemographic indicators in Census block groups in 3 study regions: Changes in greenspace metrics for an IQR increase in explanatory variables (*n*=2,285).

Table S8. Sensitivity analysis without considering air pollution for odds ratios of having an all-cause standardized mortality rate (SMR) ≥the 75th percentile mortality rate in relation to greenspace metrics for the study ZCTAs (n=169).

Table S9. Sensitivity analysis using the mortality rates in the period (2008-2013) matched with the greenness metrics: Odds ratios of having an all-cause standardized mortality rate (SMR) ≥the 75th percentile mortality rate in relation to greenspace metrics for the study ZCTAs (n=169).

Figure S1. Study areas with 3 urban centers including New Haven, CT; Baltimore, MD; and Durham, NC.

Figure S2. Comparison of greenspace metrics among quartile groups of sociodemographic indicators. (A) EVI, (B) percent tree cover, and (C) percent tree cover along walkable roads (n=2,285).

Figure S3. Odds ratios of SMR for being >the 75^th^ percentile mortality rate, in relation to greenspace metrics (*n*=169) for (A) death from circulatory disease, (B) respiratory disease, (C) cancer, (D) renal disease, and (E) mental disorders.

Figure S4. Scatter plot for the annual EVI (x-axis) and the EVI levels in the growing season (y-axis) for each census block group and year in 2008-2013.

Figure S5. Scatter plots and correlations of EVI and sociodemographic variables at the ZCTA level.

Table S1. Municipalities included in the 3 urban areas, as defined by EPA’s EnviroAtlas.

| *Urban area* | *Included Municipalities* |
| --- | --- |
| Durham, NC area (referred to as “Durham” in the main text) | Durham  Chapel Hill  Most of Carrboro  Most of Hillsborough |
| New Haven, CT area (referred to as “New Haven” in the main text | Branford  Clinton  East Haven  Essex  Guilford  Hamden  Madison  Meriden  New Haven  North Branford  North Haven  Old Saybrook  Orange  Wallingford  West Haven  Some of Cheshire  Some of Chester  Some of Deep River  Some of Durham  Some of Middlefield  Some of Westbrook |
| Baltimore, MD area (referred to as “Baltimore” in the main text) | Annapolis  Baltimore  Highland Beach |

Table S2. Definitions and data sources for the exposure and outcome variables and covariates.

| Category | Variable | Definition | Data source |
| --- | --- | --- | --- |
| Greenspace metrics | Annual average EVI | Average EVI value for all days in the year 2008–2013 | Moderate Resolution Imaging Spectroradiometer (MODIS) product MOD13Q1 by the NASA’s Earth Observing System |
|  | Seasonal EVI | Average EVI value through May–October in the year 2008­–2013 |  |
|  | Percent greenspace | Percent green space refers to the percentage of land covered by vegetation or greenspace (trees, lawns, gardens, crop land, forests, wetlands) for a given spatial unit (e.g., census block groups, ZCTA) | EnviroAtlas Database by the US Environmental Protection Agency (EPA) |
|  | Percent tree cover | Percent of total land covered by trees including street trees, parks, forests, and single trees for a given spatial unit (e.g., census block groups, ZCTA) |  |
|  | Percent of people living <500 of a park entrance | Percent of population living within a walking distance <500m from a park entrance (state, county, or local park) for a given spatial unit (e.g., census block group, ZCTA). EPA designated the park entrance for every 0.50 kilometer along the border of a given park that was open to the street to estimate this index. |  |
|  | Percent tree cover along walkable roads | Percent of tree cover along walkable roads, that were defined as roads with a speed limit <55 miles/hour, for a given spatial unit (e.g., census block group, ZCTA) |  |
| Health outcome | All cause age-standardized mortality rate | Age-standardized mortality rate from all-cause death (International Classification of Diseases [ICD] version 10 code: A00–R99) | Connecticut Department of Public health; North Carolina Division of Public Health; Maryland Department of Health |
|  | Age-standardized mortality rate from circulatory causes | Age-standardized mortality rate from circulatory causes (ICD-10 code: I00–I99) |  |
|  | Age-standardized mortality rate from respiratory causes | Age-standardized mortality rate from respiratory causes (ICD-10 code: J00–J99) |  |
|  | Age-standardized mortality rate from cancers | Age-standardized mortality rate from cancers (ICD-10 code: C00–D49) |  |
|  | Age-standardized mortality rate from renal diseases | Age-standardized mortality rate from renal diseases (ICD-10 code: N00–N39) |  |
|  | Age-standardized mortality rate from mental disorders | Age-standardized mortality rate from mental disorders (ICD-10 code: F01–F99) |  |
| Sociodemographic indicator | Percent of low-income people | Percent of population in households with household income < twice the federal poverty level | EJSCREEN Database by the US EPA |
|  | Percent of people of color | The percent of individuals who list their racial status as a race other than White alone and/or list their ethnicity as Hispanic or Latino (i.e., all people other than non-Hispanic White-alone individuals). The word "alone" in this case indicates that the person is of a single race, not multiracial. This index was originally estimated for census block groups. |  |
|  | Percent ≥25 years with less than high school education | Percent of people age 25 or older in a block group whose education is short of a high school diploma |  |
|  | Percent of living in linguistically isolated households | Percent of people in a block group living in linguistically isolated households. A household in which all members age 14 years and over speak a non-English language and also speak English less than "very well" (have difficulty with English) is linguistically isolated. |  |
|  | Percent of population <5 years | Percent of people in a block group under the age of 5 years |  |
|  | Percent of population >64 years | Percent of people in a block group over the age of 64 years |  |
| Air pollution | Average PM_2.5_ | Annual concentration of PM_2.5_ concentrations from 1-km resolution gridded modeling data averaged for each block group for the year 2012 | Air pollution modeling data from the Socioeconomic Data and Application Center |
|  | Average O_3_ | Annual concentration of O_3_ concentrations from 1-km resolution gridded modeling data averaged for each block group for the year 2012 |  |

Table S3. Odds ratios of SMR for being ≥the 75^th^ percentile mortality rate, in relation to greenspace metrics (*n*=169).

| Greenspace metrics | OR (95% CI) | | | | |
| --- | --- | --- | --- | --- | --- |
|  | Circulatory mortality | Respiratory mortality | Mortality from cancers | Mortality from renal disease | Mortality from mental disorders |
| Annual EVI (IQR=0.08) | 1.282 (0.444, 3.700) | 0.847 (0.326, 2.199) | 1.202 (0.409, 3.532) | 1.017 (0.323, 3.197) | 0.627 (0.261, 1.508) |
| Seasonal EVI (IQR=0.12) | 0.930 (0.299, 2.894) | 0.665 (0.229, 1.929) | 1.058 (0.329, 3.400) | 0.848 (0.246, 2.931) | 0.449 (0.167, 1.205) |
| Percent greenspace (%) (IQR=28.1) | 0.027 (0.004, 0.182) | 0.141 (0.035, 0.573) | 0.145 (0.032, 0.658) | 0.127 (0.025, 0.651) | 0.222 (0.066, 0.743) |
| Percent tree cover (%) (IQR=26.6) | 0.011 (0.001, 0.111) | 0.080 (0.014, 0.447) | 0.101 (0.015, 0.675) | 0.083 (0.012, 0.593) | 0.191 (0.046, 0.801) |
| Percent of people living within 500m of a park entrance (%) (IQR=21.1) | 1.235 (0.508, 3.002) | 1.203 (0.486, 2.979) | 1.382 (0.454, 4.213) | 1.233 (0.524, 2.903) | 1.863 (0.916, 3.79) |
| Percent tree cover along walkable roads (%) (IQR=19.6) | 0.088 (0.019, 0.399) | 0.190 (0.054, 0.666) | 0.295 (0.077, 1.128) | 0.368 (0.095, 1.426) | 0.402 (0.138, 1.172) |

Note. All models were adjusted for annual mean PM_2.5_ and O_3_ concentrations, percent of low-income people, percent of persons who are people of color, percent of individuals with less than high school education, percent of individuals with linguistic isolation, population density, coordinates of the centroid of ach ZCTA, and an indicator variable for each study area.

Table S4. Regression analysis for greenspace metrics and sociodemographic indicators in Census block groups in 3 study regions, separately.

| Variable | Beta (95% CI) | | | | |
| --- | --- | --- | --- | --- | --- |
|  | EVI (annual) | Percent greenspace | Percent tree cover | Percent of people living within 500m of a park entrance | Percent tree cover along walkable roads |
| New Haven, CT area (*n* = 444) | | | | | |
| Percent of persons who are people of color (IQR=64.7) | -0.019 (-0.037, -0.001) | -0.1 (-4.6, 4.5) | 0.0 (-5.7, 5.8) | 10.4 (-0.8, 21.5) | 0.7 (-4.4, 5.8) |
| Percent of low income (%) (IQR=33.6) | -0.028 (-0.04, -0.016) | -6.6 (-9.6, -3.6) | -7.5 (-11.3, -3.7) | 9.3 (1.9, 16.6) | -5.8 (-9.2, -2.5) |
| Percent of linguistic isolation (IQR=3.8) | -0.005 (-0.008, -0.002) | -1.0 (-1.8, -0.2) | -0.9 (-1.9, 0.1) | -0.1 (-2.1, 1.8) | -0.2 (-1.1, 0.7) |
| Percent of people with less than high school education (IQR=15.5) | -0.003 (-0.013, 0.007) | -2.9 (-5.5, -0.3) | -3.0 (-6.3, 0.3) | 0.0 (-6.4, 6.4) | -4.8 (-7.7, -1.9) |
| Percent of population under 5 years (IQR=5.3) | 0.003 (-0.004, 0.01) | 0.0 (-1.7, 1.8) | -0.3 (-2.5, 1.9) | -1.1 (-5.3, 3.2) | -0.1 (-2.1, 1.8) |
| Percent of population over 64 years (IQR=10.7) | -0.007 (-0.013, -0.001) | 0.5 (-1.1, 2.0) | -1.0 (-2.9, 0.9) | -4.6 (-8.3, -0.9) | -0.2 (-1.9, 1.5) |
| Population density (people/km^2^) (IQR=3070.6) | -0.034 (-0.042, -0.027) | -9.7 (-11.7, -7.8) | -5.6 (-8.0, -3.1) | 8.3 (3.5, 13.1) | -4.1 (-6.3, -1.9) |
| Baltimore, MD area (n = 1,648) | | | | | |
| Percent of persons who are people of color (IQR=64.7) | 0.004 (-0.003, 0.012) | -2.6 (-4.5, -0.7) | -1.1 (-2.9, 0.7) | -0.5 (-4.7, 3.7) | -2.8 (-4.5, -1.0) |
| Percent of low income (%) (IQR=33.6) | -0.031 (-0.038, -0.024) | -7.5 (-9.3, -5.7) | -4.7 (-6.3, -3.0) | 5.1 (1.2, 9.1) | -2.2 (-3.9, -0.6) |
| Percent of linguistic isolation (IQR=3.8) | 0.002 (0.000, 0.005) | 0.4 (-0.3, 1.0) | 0.2 (-0.5, 0.8) | -0.8 (-2.2, 0.7) | -0.2 (-0.8, 0.4) |
| Percent of people with less than high school education (IQR=15.5) | -0.016 (-0.021, -0.011) | -3.9 (-5.2, -2.6) | -4.9 (-6.2, -3.6) | 0.9 (-2.0, 3.8) | -6.0 (-7.2, -4.8) |
| Percent of population under 5 years (IQR=5.3) | 0.008 (0.004, 0.011) | 1.7 (0.8, 2.7) | 1.0 (0.1, 1.9) | -3.0 (-5.1, -0.9) | 0.6 (-0.2, 1.5) |
| Percent of population over 64 years (IQR=10.7) | 0.004 (0.001, 0.007) | 0.8 (-0.1, 1.6) | 1.5 (0.6, 2.3) | -2.1 (-4.0, -0.2) | 2.3 (1.6, 3.1) |
| Population density (people/km^2^) (IQR=3070.6) | -0.031 (-0.035, -0.028) | -12.8 (-13.7, -12.0) | -7.6 (-8.4, -6.8) | 11.8 (10.0, 13.7) | -4.1 (-4.8, -3.3) |
| Durham, NC area (n = 193) | | | | | |
| Percent of persons who are people of color (IQR=64.7) | 0.010 (-0.009, 0.028) | -0.3 (-6.3, 5.8) | -1.9 (-9.2, 5.4) | -12.3 (-27.4, 2.7) | -18.4 (-26.7, -10.2) |
| Percent of low income (%) (IQR=33.6) | -0.015 (-0.024, -0.006) | -5 (-7.8, -2.3) | -4.5 (-7.9, -1.2) | 9.1 (2.1, 16.0) | -2.3 (-6.1, 1.5) |
| Percent of linguistic isolation (IQR=3.8) | -0.001 (-0.003, 0.002) | -0.5 (-1.3, 0.4) | -0.6 (-1.7, 0.4) | -3.6 (-5.7, -1.5) | -0.8 (-1.9, 0.4) |
| Percent of people with less than high school education (IQR=15.5) | -0.008 (-0.017, 0.002) | -0.3 (-3.3, 2.6) | -0.5 (-4.2, 3.1) | 12.2 (4.7, 19.7) | 4.6 (0.5, 8.7) |
| Percent of population under 5 years (IQR=5.3) | 0.006 (0.000, 0.012) | 1.7 (-0.2, 3.7) | 1.6 (-0.7, 4.0) | 1.3 (-3.6, 6.1) | 0.4 (-2.2, 3.1) |
| Percent of population over 64 years (IQR=10.7) | -0.003 (-0.01, 0.003) | -1.4 (-3.5, 0.7) | -1.2 (-3.7, 1.4) | 0.9 (-4.4, 6.1) | -0.5 (-3.4, 2.4) |
| Population density (people/km^2^) (IQR=3070.6) | -0.054 (-0.07, -0.038) | -18.0 (-23.0, -13.0) | -18.9 (-24.9, -12.8) | 19.1 (6.6, 31.6) | -1.3 (-8.1, 5.6) |

Table S5. Odds ratios of all-cause standardized mortality rate (SMR) for being ≥the 75th percentile mortality rate in relation to sociodemographic variables in ZCTAs (n=169) from the model considering each greenspace metric.

| Variable | OR (95% CI) | | | | |
| --- | --- | --- | --- | --- | --- |
|  | Model containing annual EVI | Model containing percent greenspace | Model containing percent tree cover | Model containing percent of people living within 500m of a park entrance | Model containing percent tree cover along walkable roads |
| Annual mean PM_2.5_ (μg/m^3^) (IQR=1.5) | 2.05 (0.42, 10.08) | 0.84 (0.13, 5.52) | 0.68 (0.1, 4.59) | 1.85 (0.39, 8.85) | 0.96 (0.17, 5.4) |
| Annual mean O_3_ (ppb) (IQR=2.4) | 0.33 (0.12, 0.89) | 0.54 (0.19, 1.52) | 0.55 (0.19, 1.6) | 0.38 (0.16, 0.93) | 0.5 (0.19, 1.34) |
| Percent of low income (%) (IQR=19.1) | 1.10 (0.33, 3.70) | 2.30 (0.11, 48.43) | 2.42 (0.2, 29.17) | 1.11 (0.31, 3.95) | 1.62 (0.42, 6.35) |
| Percent of people of color (IQR=31.6) | 0.55 (0.14, 2.10) | 0.66 (0.13, 3.33) | 0.63 (0.14, 2.83) | 0.62 (0.17, 2.31) | 0.66 (0.17, 2.52) |
| Percent of linguistic isolation (IQR=2.1) | 0.31 (0.14, 0.65) | 0.19 (0.07, 0.51) | 0.16 (0.06, 0.47) | 0.30 (0.14, 0.63) | 0.25 (0.11, 0.58) |
| Percent of people with less than high school education (IQR=8.4) | 13.11 (3.09, 55.75) | 5.49 (0.77, 38.94) | 4.33 (0.66, 28.24) | 9.81 (2.43, 39.59) | 6.13 (1.41, 26.62) |
| Percent of population under 5 years (IQR=3.5) | 1.26 (0.40, 3.93) | 28.09 (3.45, 228.94) | 27.89 (3.8, 204.5) | 1.24 (0.4, 3.88) | 3.8 (1.04, 13.86) |
| Percent of population over 64 years (IQR=6.8) | 0.33 (0.14, 0.77) | 2.04 (0.62, 6.72) | 1.35 (0.47, 3.85) | 0.34 (0.15, 0.77) | 0.98 (0.34, 2.81) |
| Population density (people/km^2^) (IQR=1001.3) | 0.88 (0.38, 2.07) | 0.45 (0.16, 1.21) | 0.56 (0.22, 1.44) | 0.78 (0.35, 1.76) | 0.79 (0.34, 1.80) |

Table S6. Regression analysis for EVI in the growing season and sociodemographic indicators in Census block groups in 3 study regions (n=2,285).

| Variable | Beta (95% CI) |
| --- | --- |
|  | EVI in the growing season |
| Percent of persons who are people of color (IQR=64.7) | 0.004 (-0.005, 0.012) |
| Percent of low income (%) (IQR=33.6) | -0.039 (-0.046, -0.032)* |
| Percent of linguistic isolation (IQR=3.8) | 0.000 (-0.003, 0.002) |
| Percent of people with less than high school education (IQR=15.5) | -0.020 (-0.026, -0.014)* |
| Percent of population under 5 years (IQR=5.3) | 0.007 (0.003, 0.011)* |
| Percent of population over 64 years (IQR=10.7) | 0.002 (-0.002, 0.006) |
| Population density (people/km^2^) (IQR=3070.6) | -0.048 (-0.052, -0.044)* |

Table S7. Sensitivity analysis of greenspace metrics and sociodemographic indicators in Census block groups in 3 study regions: Changes in greenspace metrics for an IQR increase in explanatory variables (*n*=2,285).

| Variable | Beta (95% CI) | | | | |
| --- | --- | --- | --- | --- | --- |
|  | EVI (annual) | Percent greenspace | Percent tree cover | Percent of people living within 500m of a park entrance | Percent tree cover along walkable roads |
| Percent of Black/African American people (IQR=55.2) | 0.005 (0.000, 0.010)* | -1.4 (-2.7, -0.2)* | 0.1 (-1.2, 1.4) | -0.7 (-3.5, 2.2) | -0.8 (-2.0, 0.4) |
| Percent of low income (%) (IQR=33.6) | ­-0.031 (-0.036, -0.026)* | -7.5 (-8.8, -6.1)* | -5.2 (-7.5, -4.8)* | 6.5 (3.4, 9.5) | -3.7 (-5.1, -2.3)* |
| Percent of linguistic isolation (IQR=3.8) | 0.000 (–0.001, 0.002) | 0.1 (-0.4, 0.6) | -0.1 (-0.5, 0.4) | -0.3 (-1.4, 0.7) | -0.1 (-0.6, 0.3) |
| Percent of people with less than high school education (IQR=15.5) | -0.015 (-0.019, -0.011)* | -3.7 (-4.7, -2.6) | -4.1 (-5.2, -3.0)* | 2.2 (-0.3, 4.6) | -5.2 (-2.3, -4.1)* |
| Percent of population under 5 years (IQR=5.3) | 0.005 (0.002, 0.008)* | 1.2 (0.4, 1.9) | 0.7 (-0.1, 1.4) | -2.3 (-4.0, -0.5)* | 0.2 (-0.6, 0.9) |
| Percent of population over 64 years (IQR=10.7) | 0.001 (-0.002, 0.004) | 0.2 (-0.5, 0.9) | 0.7 (-0.1, 1.4) | -2.7 (-4.3, -1.1)* | 1.7 (1.0, 2.4)* |
| Population density (people/km^2^) (IQR=3070.6) | -0.036 (-0.038, -0.033)* | -13.5 (-14.3, -12.8) | -8.4 (-9.1, -7.6)* | 11.4 (9.7, 13.0)* | -4.9 (-5.6, -4.1)* |

Note. All models were adjusted for the centroids of each block group and an indicator variable of study regions.

*Significant at a significance level of 0.05.

Table S8. Sensitivity analysis without considering air pollution for odds ratios of having an all-cause standardized mortality rate (SMR) ≥the 75^th^ percentile mortality rate in relation to greenspace metrics for the study ZCTAs (*n*=169).

| Variable | OR (95% CI) |
| --- | --- |
| Annual EVI (IQR=0.08) | 0.72 (0.28, 1.8) |
| EVI in growing season (IQR=0.12) | 0.48 (0.18, 1.33) |
| Percent greenspace (%) (IQR=28.1) | 0.02 (0.00, 0.11)* |
| Percent tree cover (%) (IQR=26.6) | 0.01 (0.00, 0.06)* |
| Percent of people living within 500m of a park entrance (%) (IQR=21.1) | 1.57 (0.61, 4.03) |
| Percent tree cover along walkable roads (%) (IQR=19.6) | 0.06 (0.01, 0.32)* |

Note. All models were adjusted for the centroids of each block group, an indicator variable of study regions, percent of low-income people, percent of people who are people of color, percent of linguistic isolation, percent of people with less than high school education, Percent of population under 5 years, Percent of population over 64 years, and population density.

*Significant at a significance level of 0.05.

Table S9. Sensitivity analysis using the mortality rates in the period (2008-2013) matched with the greenness metrics: Odds ratios of having an all-cause standardized mortality rate (SMR) ≥the 75^th^ percentile mortality rate in relation to greenspace metrics for the study ZCTAs (*n*=169).

| Variable | OR (95% CI) |
| --- | --- |
| Annual EVI (IQR=0.08) | 0.94 (0.31, 2.90) |
| EVI in growing season (IQR=0.12) | 0.70 (0.21, 2.34) |
| Percent greenspace (%) (IQR=28.1) | 0.04 (0.01, 0.22)* |
| Percent tree cover (%) (IQR=26.6) | 0.04 (0.00, 0.29)* |
| Percent of people living within 500m of a park entrance (%) (IQR=21.1) | 1.61 (0.61, 4.27) |
| Percent tree cover along walkable roads (%) (IQR=19.6) | 0.22 (0.05, 0.86)* |

Note. All models were adjusted for the centroids of each block group, an indicator variable of study regions, annual mean PM_2.5_ and O_3_ concentrations, percent of low-income people, percent of people who are people of color, percent of linguistic isolation, percent of people with less than high school education, Percent of population under 5 years, Percent of population over 64 years, and population density.

*Significant at a significance level of 0.05.


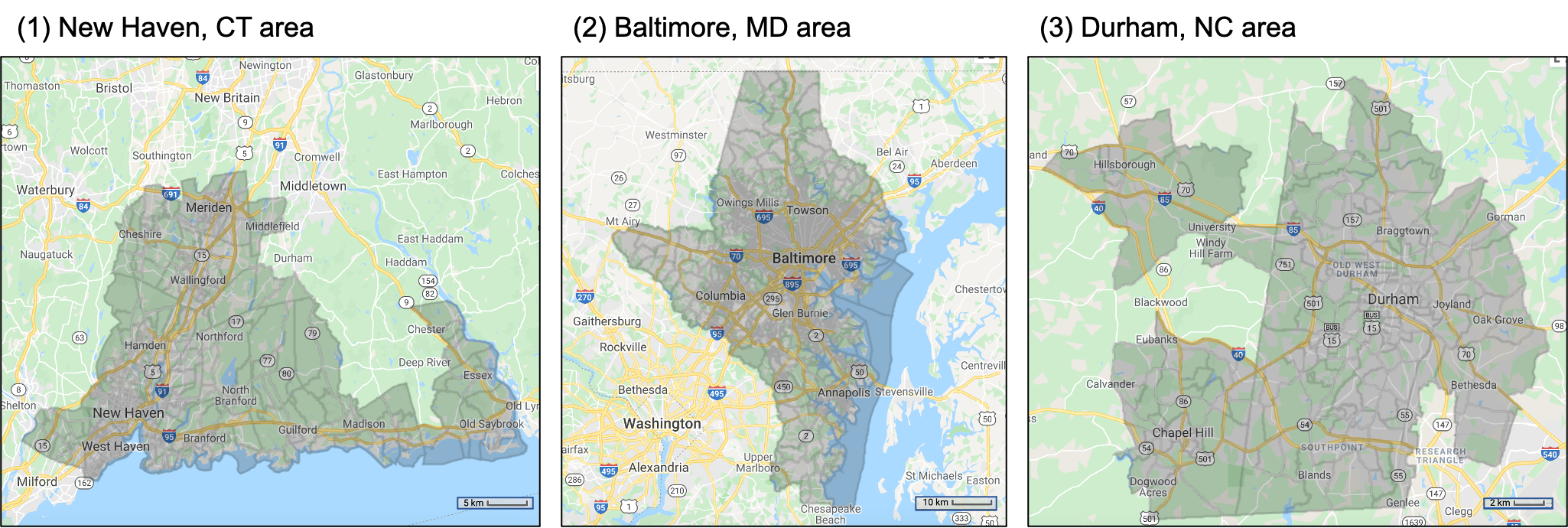


Figure S1. Study areas with 3 urban centers including New Haven, CT; Baltimore, MD; and Durham, NC.


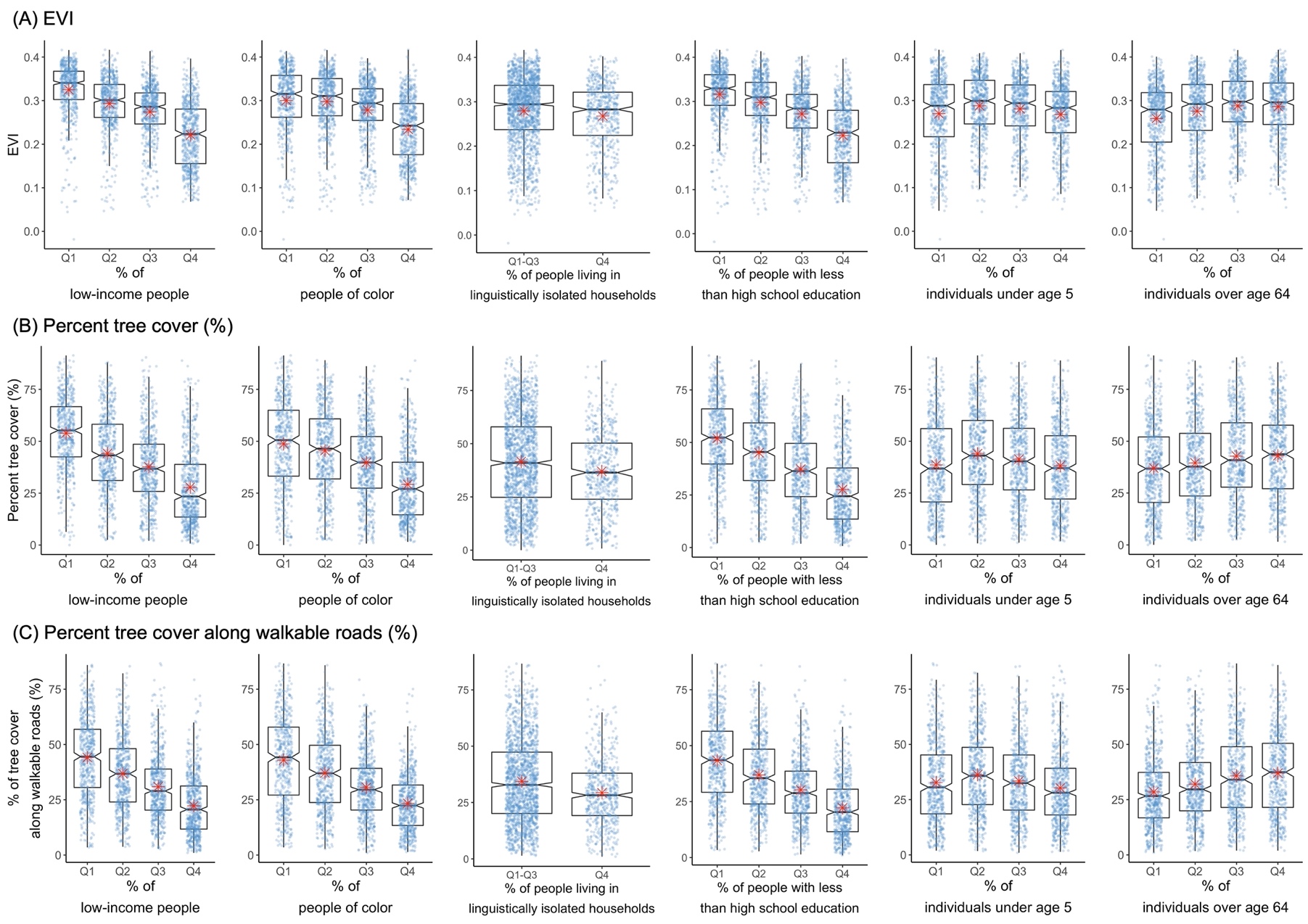


Figure S2. Comparison of greenspace metrics among quartile groups of sociodemographic indicators. (A) EVI, (B) percent tree cover, and (C) percent tree cover along walkable roads (n=2,285).

Note. Red asterisk indicates the average within each group. Blue points indicate value of greenspace metrics in each block group.


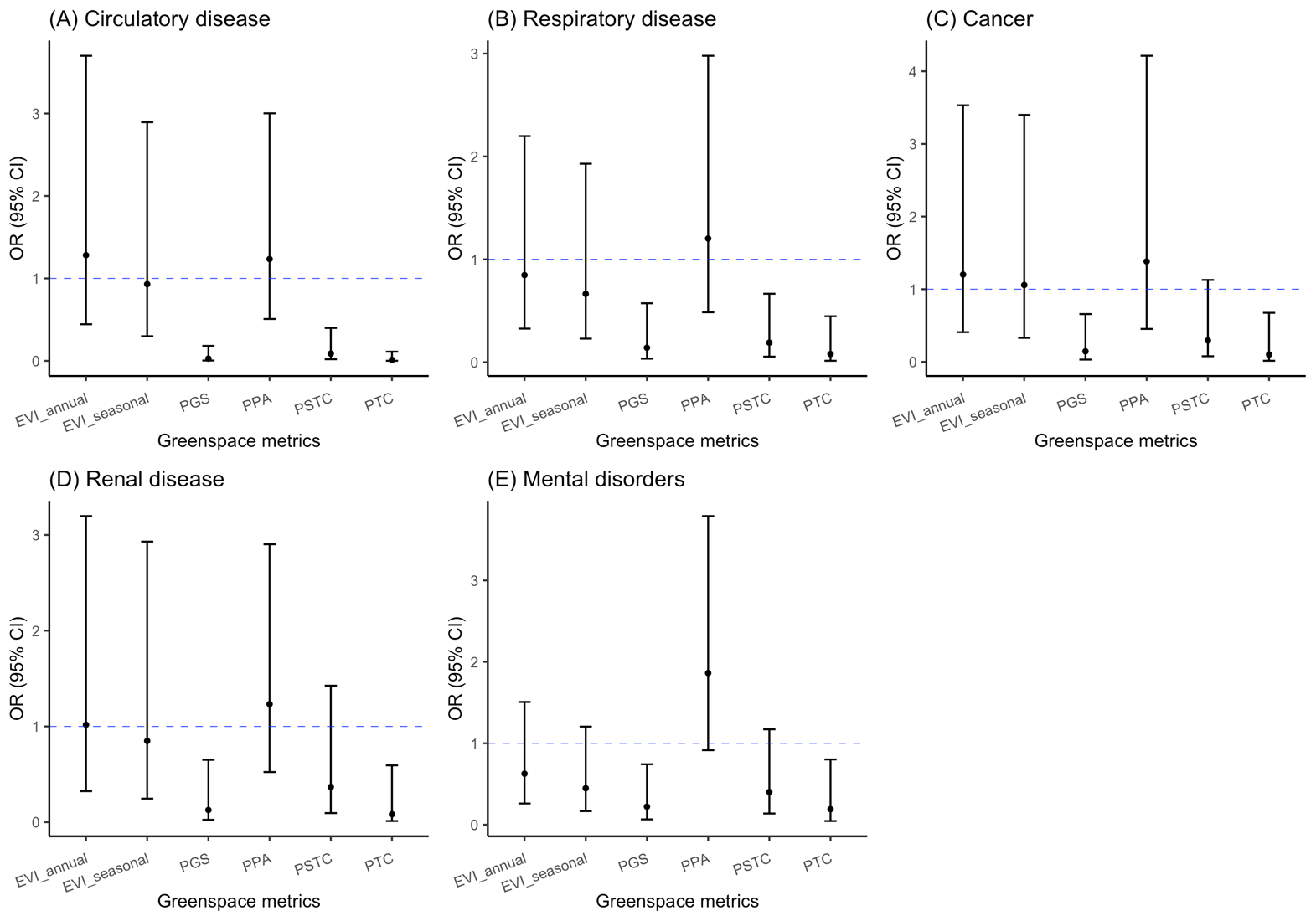


Figure S3. Odds ratios of SMR for being >the 75^th^ percentile mortality rate, in relation to greenspace metrics (*n*=169) for (A) death from circulatory disease, (B) respiratory disease, (C) cancer, (D) renal disease, and (E) mental disorders. EVI: Enhanced Vegetation Index, PGS: percent greenspace, PPA: percent of people living within 500m of a park entrance, PSTC: Percent tree cover along with walkable roads, PTC: percent tree cover.


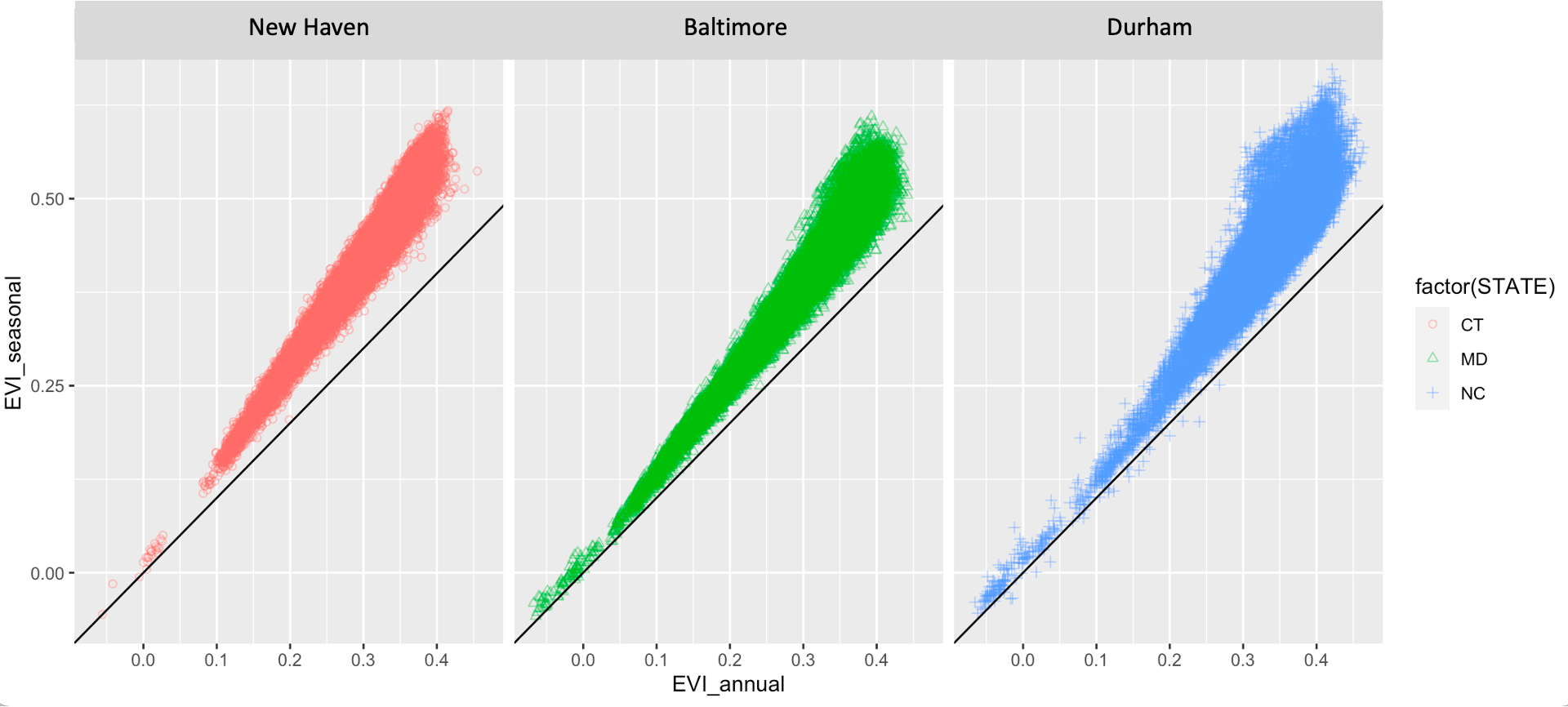


Figure S4. Scatter plot for the annual EVI (x-axis) and the EVI levels in the growing season (y-axis) for each census block group and year in 2008-2013.





Figure S5. Scatter plots and correlations of EVI and sociodemographic variables at the ZCTA level.
